# Supplementary material for: Complex Network Geometry and Frustrated Synchronization
Source: Sci Rep. 2018 Jul 2;8:9910. doi: 10.1038/s41598-018-28236-w (PMC6028575; doi:10.1038/s41598-018-28236-w)
Supplement: Supplementary file 1 — Supplementary Information [file 41598_2018_28236_MOESM1_ESM.pdf]

Supplementary Material for "Complex Network Geometry and Frustrated Synchronization"

Ana P. Millán[1], Joaquín J. Torres[1], and Ginestra Bianconi[2]

[1]Departamento de Electromagnetismo y Física de la Materia and Instituto Carlos I de Física Teórica y Computacional, Universidad de Granada, 18071 Granada, Spain
[2]School of Mathematical Sciences, Queen Mary University of London, E1 4NS London, United Kingdom

ABSTRACT

In this Supplementary Material we provide additional information about the structure of Complex Network Manifolds regarding its degree distribution and its small-word character. Moreover we report additional information on the Complex Network Manifolds that have been used in the movies included as further Supplementary Material of this work.

1 Degree distribution and Hausdorff dimension of Complex Network Manifolds

The degree distribution $p(k)$ of Complex Network Manifold[1,2] is exponential for dimension $d=2$ and scale-free for $d>2$. The exact asymptotic expression has been derived in Ref.[3] and is given for $d=2$ by

$$p(k)=\frac{1}{d+1}\left(\frac{2}{3}\right)^{k-d}, \tag{S-1}$$

with $k\geq 2$ whereas for $d>2$ it is given by

$$p(k)=\frac{d-1}{2d-1}\frac{\Gamma[(1+(2d-1)/(d-2)]}{\Gamma[d/(d-2)]}\frac{\Gamma[k-d+d/(d-2)]}{\Gamma[k-d+1+(2d-1)/(d-2)]}, \tag{S-2}$$

with $k\geq d$. Therefore, for $d>2$ Complex Network Manifolds are scale-free with a power-law scaling

$$p(k)\approx k^{-\gamma}, \tag{S-3}$$

valid for $k\gg 1$, and power-law exponent γ

$$\gamma=2+\frac{1}{d-2}. \tag{S-4}$$

Therefore, for $d=3$ we obtain $\gamma=3$ and for $d=4$ we obtain $\gamma=5/2=2.5$. In Fig. 1S (a) we show the agreement between the analytic expression (dashed lines) and the computational results (data points).

The logarithmic scaling of the average shortest (hopping) distance ℓ between the nodes of the network with the network size N, is known to reveal the small-world[4] nature of a network. By investigating numerically the scalling of ℓ with N we show that Complex Network Manifolds are small world. Our result are reported in Fig.1S (b) where points represent data from numerical simulations of Complex Network Manifold of dimension d, whereas the solid lines stand for the best logarithmic fit, as given by

$$\ell=a_d\log(N)+b_d. \tag{S-5}$$

The parameters from the fit are shown in the Table $S-I$, and they clearly indicate that the Complex Network Manifolds of higher dimension d have a average shortest distance that grows always logarithmically with the network size N but with different constant prefactor a_d.

d	a_d	b_d	R^2
2	2.93(3)	−1.45(9)	0.983
3	1.32(2)	0.17(4)	0.964
4	0.78(1)	0.79(4)	0.954

Table S-I. Fitted parameters a_d and b_d determining the logarithmic growth of the average shortest (hopping) distance ℓ of Complex Network Manifolds in dimension d according to Eq. $(S-5)$.

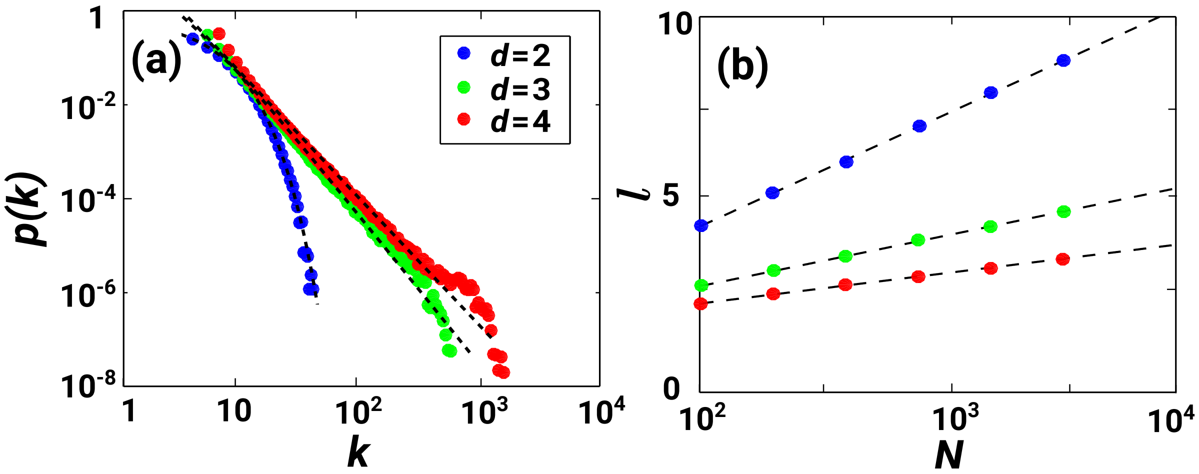

Supplementary Figure 1S. Degree distribution and small-word properties of Complex Network Manifolds. The degree distribution $p(k)$ of the Complex Network Manifolds of $N = 6400$ nodes and dimensions $d = 2,3$ and 4 is plotted in panel (a). Points represent results from numerical simulations whereas dashed lines stand for the analytical result as given by eq. S-1 and S-2. The network diameter D is plotted versus the network size N for dimensions $d = 2,\ 3,\ 4$ in panel (b). Data points are from simulation results whereas dashed lines correspond to the logarithmic fit. Numeric results have been averaged over 100 network realizations in both plots.

2 Complex Network Manifolds that have been used for the movies of temporal activity

Further Supplementary Materials include the movies for the temporal activity of three Complex Network Manifolds in $D = 1, D = 2$ and $D = 3$ respectively. These networks have $N = 200$ nodes and random assignment of their internal frequencies. The activity of the nodes is recorded for different values of the coupling constant σ by coloring the nodes according to a color code depending on $\cos(\theta)$.

In Figure 2*S* as a reference we plot the $R(T)$ curve as a function of the coupling constant σ as recorded for the Complex Network Manifolds captured by the movies.

References

1. Bianconi G., & Rahmede, C. Complex quantum network manifolds in dimension $d > 2$ are scale-free. *Scientific Reports* **5**, 13979 (2015).
2. Bianconi, G. & Rahmede, C. Network geometry with flavor: from complexity to quantum geometry. *Phys. Rev. E* **93**, 032315 (2016).
3. Bianconi G. & Rahmede, C. Emergent hyperbolic network geometry. *Scientific Reports* **7** (2017).
4. Watts, D. J. & Strogatz, S. H. Collective dynamics of 'small-world'networks. *Nature* **393**, 440 (1998).

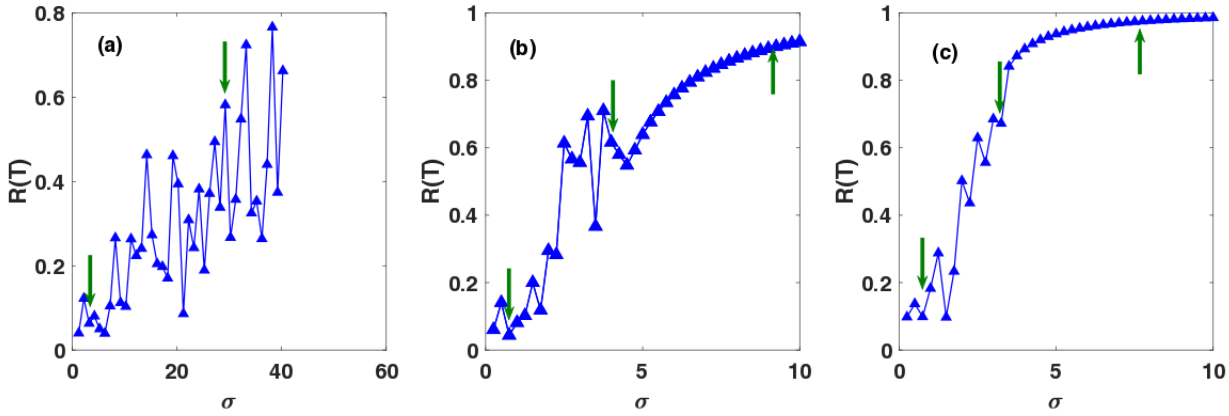

Supplementary Figure 2S. Frustrated synchronization for the Complex Network Manifolds shown in the movies
The synchronization order parameter $R(T)$ is plotted versus the coupling strength σ for $D = 1$ (a), $D = 2$ (b) and $D = 3$ (c), for a single network realization of $N = 200$ nodes. The arrows indicate the coupling constants σ at which the movies are recorded (for $D = 1$, $\sigma = 3, 29$ for $D = 2$ $\sigma = 0.75, 4.00, 8.74$ for $D = 3$ $\sigma = 0.75, 3.25, 7.25$).
